# Daytime versus nighttime laparoscopic appendectomy in term of complications and clinical outcomes: A retrospective study of 1001 appendectomies

**DOI:** 10.1016/j.heliyon.2022.e11911

**Published:** 2022-11-30

**Authors:** Amjad A. Shah, Raed M. Al-Zoubi, Ahmad R. Al-Qudimat, Mohamed Amine Rejeb, Laxmi Kumari Ojha, Sharif Abdulzem, Khadija Qadir, Sara Sameer, Ahmad Zarour, Mohamed Said Ghali

**Affiliations:** a Acute Care Surgery Division, Department of Surgery, Hamad Medical Corporation, Doha, Qatar; b Surgical Research Section, Department of Surgery, Hamad Medical Corporation, Doha, Qatar; c Department of Chemistry, Jordan University of Science and Technology, P.O. Box 3030, Irbid, 22110, Jordan; d Department of Biomedical Sciences, QU-Health, College of Health Sciences, Qatar University, Doha, 2713, Qatar; e Department of Public Health, Qatar University, Doha, Qatar; f Department of General Surgery, Ain Shams University Cairo, Egypt

**Keywords:** Laparoscopic, Appendectomy, Outcomes, Shifts, Complications, Delay

## Abstract

**Purpose:**

This research aims to study whether the time of day impacts the outcome of laparoscopic appendectomy. Peri/post-operative data including type of surgery, operating room time, length of stay, re-hospitalization rates, and short/long term morbidity and mortality were assembled and analyzed.

**Methods:**

A retrospective review of all patient's charts who underwent an appendectomy for acute appendicitis at the Acute Care Surgery division at Hamad Medical Corporation (HMC) from December 2017 to July 2020 was performed. Our institution implemented SAGES protocol to patients with laparoscopic appendectomy. Medical history, symptoms, duration of symptoms, type of treatment, complication, experience level of surgeons in different shift, morbidity, mortality, and length of hospital stay were extracted and analyzed.

**Results:**

Multivariate logistic regression analysis was used to identify the odds ratio (OR) and the correlation of variables with different surgical groups. A total of 1001 patients were included in this study and underwent laparoscopic appendectomy, 51.3% were operated during the daytime shifts and 48.7% during the nighttime shifts. The majority of surgeries were operated during the nighttime shift C (1:00 a.m. to 7:00 a.m.). Neither there was any difference in clinical outcomes nor day/night operation time with physicians. A statistically significant correlation was found between hospital-stay of patients with different surgical group (OR: 2.13, 95% CI: 0.75–0.93, P < 0.001).

**Conclusion:**

Appendectomy conducted at night is correlated with similar complications as appendectomy performed during the day, and that the varied shift hours had no effect on the complication rates or on the quality of care provided to patients at our hospital.

## 1 Introduction

Appendicitis, defined as an inflammation of the vermiform appendix, is the predominant cause for emergency abdominal surgery, with an estimated lifetime risk reported to be 7–8%. Globally, its annual incidence is 95.5–100 cases per 100,000 adult population [1, 2, 3]. Appendicitis is thought to be caused by luminal obstruction from various etiologies, leading to increased mucus production and bacterial overgrowth, resulting in wall tension and, eventually, necrosis and potential perforation [4]. However, the full range of specific causes of appendicitis remain unknown [5].

The diagnosis of acute appendicitis is based on history, clinical presentation, laboratory tests, imaging and scoring systems such as Alvarado score [6] and Appendicitis inflammatory response (AIR) score [7]. Acute appendicitis is usually diagnosed accurately and promptly in more than 90% of cases [8]. Family history of acute appendicitis heightened roughly to more than three times the risk of appendicitis for respective family members [9].

Open appendectomy has long been the gold standard for appendicitis since the 18th century [10]. However, in the past 40 years, laparoscopy has gradually become a routine surgical treatment. Compared with an open approach, laparoscopic appendectomy is associated with less postoperative pain and faster recovery, earlier hospital discharge, and faster return to normal state of health [11, 12, 13]. It has been shown that postoperative complication rate is significantly increased among those with perforated appendicitis [14, 15].

The rate of perforated appendicitis varies between 16% to 40% with a higher frequency occurring in younger age groups [40–57%] and in patients older than 50 years [55–70%] [16].

According to Dutch guidelines, in case of suspected appendicitis, surgery should be performed within 8 h [17]. However, the timing of appendectomy remains debated. In a conducted meta-analysis report [18], it was found that the delay of surgery up to 24 h for patients with uncomplicated appendicitis was not associated with postoperative complications and surgery postponement, therefore safe in this group. Pogorelić et al. reported another meta-analysis study on the incidence of complicated appendicitis in the COVID-19 pandemic versus the pre-pandemic time [19]. The authors showed a significantly higher incidence of complicated appendicitis during the COVID-19 pandemic than in the pre-COVID-19 period, due to delay in surgery. Additionally, a significantly higher proportion of patients was managed via the non-operative approach during the pandemic in comparison to the pre-pandemic period.

Such findings could encourage the reduction of unnecessary straightforward surgeries for uncomplicated acute appendicitis and control the disruption of operating room schedules [20, 21]. Conversely, a positive association [22, 23, 24, 25, 26] was found between appendicitis suspicion time interval and the risk of perforation, as a delay of surgery resulted in complicated appendicitis and consequently, postoperative morbidity.

Laparoscopic appendectomy is the standard of care for acute appendicitis at our center, with specialized theater services accessible 24 h a day. Consultants, specialists, fellows, and residents make up the surgical team. The shift pattern includes two teams 24/7 coverage alternating between working two 12-hour shifts starting from 7:00 a.m.

This paper aims to study the effect of time of surgical intervention on the outcome of laparoscopic appendectomy and if different shifts could be associated with worse clinical outcomes. Peri/post-operative data including type of surgery, operating room time, length of stay, re-hospitalization rates, and short/long term morbidity and mortality will be assembled and analyzed.

## 2 Method

We conducted a retrospective observational study from Dec 2017 to July 2020. We included every patient (n = 1001) of age ≥18 years diagnosed with acute appendicitis, who was referred to the department of surgery, Hamad Medical Corporation HMC, Doha, Qatar. Our institution implemented SAGES protocol to patients with laparoscopic appendectomy [27]. Exclusion criteria were any admitted patient of age <18 years and patients with negative appendectomies. We extracted the data for patients who could recollect the exact timing of symptom onset, and patients who had appendicitis in the pathology examination. The diagnosis of acute appendicitis was performed by the attending surgeon on clinical grounds added by laboratory data, ultrasound, or computed tomography scan images prior to surgery in all patients. Chart review was conducted regarding the perioperative complications during 4-different working shifts of surgeons. Demographic data, medical history, Postoperative complication rate, hospital stay, and mortality rate were recorded and then stored on a spreadsheet for further analysis. Studying the safety of laparoscopic appendectomy defined by the incidence of operative complications (like bleeding, bowel injury and Surgical site infection), post-operative morbidity, and prognosis in four groups (daytime and nighttime) was the primary outcome studied in this research. The relationship between surgeons' experience, patients' clinical condition, and other data on the incidence of operative complications (like bleeding, bowel injury and Surgical site infection), post-operative morbidity, prognosis was the secondary outcome.

### 2.1 In-hospital

All surgeons in the acute care surgery division at HMC were either trainee surgical residents and fellows or attendings. The hospital work is 24h and in two shifts (Day shift from 7:00 a.m. to 7:00 p.m., and night shift from 7:00 p.m. to 7:00 a.m.) during weekdays and weekends. Appendectomies were categorized to four groups each is 6 h apart, two in the daytime shift (A and B groups) and two in nighttime shift (C and D groups).

### 2.2 Ethical aspects

The study was conducted according to the World Medical Association Declaration of Helsinki and was approved by the Medical Research Committee in HMC (MRC-01-20-722, September 02, 2020).

### 2.3 Statistical analysis

Analyses were performed using STATA-17 for windows and GraphPad Prism version 8. Chi-square tests were performed to compare categorical variables, t-test and ANOVA test for continuous variables. P-value less than 0.05 was considered a significant value. Multivariate analysis (ordinal logistic regression analysis) was used to identify the odds ratio (OR) and the correlation of variables with different surgical groups.

## 3 Results

A total of 1,001 patients were included in this study, 71% male and 29% female patients with a mean age of (32.23 ± 10.03) as described in Table 1. Among the four groups, 26.7% (n = 267) and 24.7% (n = 247) were operated during the daytime period from 7:00 a.m. to 1:00 p.m. (Group A) and from 1:00 p.m. to 7:00 p.m. (Group B) respectively. In addition, 19.4% (n = 194) from 7:00 p.m. to 1:00 a.m. (Group C) and 29.3% (n = 293) from 1:00 a.m. to 7:00 a.m. (Group D) were operated during the nighttime periods. Highest number of cases were operated during the night shift group **D** (1:00 a.m. to 7:00 a.m.). Overall, 93.5% of patients had no comorbidities, with the most common comorbidity being hypertension but with only 1.9% (n = 19) of patients. We found more than 99% of patients' pre-operative symptoms to be pain (n = 1000) and tenderness (n = 997), 71.4% (n = 715) Nausea/Vomiting, rebound tenderness in 70.7% (n = 708) and 4.7% (n = 47) for fever. The duration of symptom's days for most patients were found between 1-3 days (94.7%) with a statistically significant correlation (ordinal logistic regression analysis) between the four groups as shown in Table 1 (OR: 1.8, 95% CI: 0.76–0.96, P < 0.001).

**Table 1:** Patients baseline characteristic.

Age (Mean ± SD)	32.23 ± 10.03				
Items	Group A	Group B	Group C	Group D	P-value
	7.00 am–1.00 pm	1.00 pm–7.00 pm	7.00 pm–1.00 am	1.00 am–7.00 am	
Gender (N/%)					0.361
Male	**190**/71.2%	**166**/67.2%	**142**/73.2%	**216**/73.7%	
Female	**77**/28.8%	**81**/32.8%	**52**/26.8%	**77**/26.3%	
Total	**267** (**26.7%**)	**247** (**24.7%**)	**194** (**19.4%**)	**293** (**29.3%**)	
Nationality (N/%)					0.389
Asia	**224**/83.9%	**185**/74.9%	**155**/79.9%	**229**/78.2%	
Europe	**2**/0.7%	**4**/1.6%	**3**/1.5%	**3**/1.0%	
North America	**0**/0.0%	**1**/0.4%	**1**/0.5%	**0**/0.0%	
Africa	**40**/14.9%	**57**/23.1%	**35**/18.0%	**61**/20.8%	
Unknown	**1**/0.5%	**0**/0.0%	**0**/0.0%	**0**/0.0%	
Comorbidities (N/%)					0.912
Yes	**20**/7.5%	**13**/5.3%	**12**/6.2%	**20**/6.8%	
No	**247**/92.5%	**234**/94.7%	**182**/93.8%	**273**/93.2%	
DM	**5**/1.87%	**3**/1.21%	**2**/1.03%	**2**/0.68%	0.191
DM & HTN with others	**2**/0.75%	**4**/1.62%	**3**/1.55%	**5**/1.71%	
HTN	**5**/1.87%	**2**/0.81%	**5**/2.58%	**7**/2.39%	
Others	**8**/3.0%	**4**/1.62%	**2**/1.03%	**6**/2.05%	
Symptoms (N/%)					
Pain	**267**/100%	**247**/100%	**194**/100%	**292**/99%	0.490
Fever	**12**/4.5%	**9**/3.6%	**14**/7.2%	**12**/4.1%	0.915
Nausea/vomiting	**184**/68.9%	**189**/76.5%	**138**/71.1%	**204**/69.6%	0.218
Tenderness	**266**/99%	**247**/100%	**192**/99%	**292**/99%	0.397
Rebound	**179**/67%	**184**/74.5%	**137**/70.6%	**208**/71.0%	0.327
Duration of symptoms' days (N/%)					<0.001
1	**164**/61.4%	**142**/57.5%	**120**/61.9%	**169**/57.7%	
2	**59**/22.1%	**62**/25.1%	**52**/26.8%	**84**/28.7%	
3	**29**/10.9%	**29**/11.7%	**13**/6.7%	**25**/8.5%	
4	**7**/2.6%	**8**/3.2%	**6**/3.1%	**9**/3.1%	
5	**2**/0.75%	**2**/0.81%	**2**/1.0%	**4**/1.4%	
6	**2**/0.75%	**0**/0.0%	**1**/0.52%	**0**/0.0%	
7	**4**/1.5%	**4**/1.6%	**0**/0.0%	**2**/0.68%	
Mean (SD)	**1.6 (1.0)**	**1.6 (1.0)**	**1.7 (1.1)**	**1.7 (1.1)**	

Intra and postoperative outcomes for four different in-hospital groups are represented in Table 2. The most common operative finding among all groups was inflamed appendix with 99.6% (n = 997, P = 0.604). Others were also reported such as mass, abscess, perforated and gangrenous giving 6.5% (P = 0.636), 3.0% (P = 0.973), 5.5% (P = 0.0.302), and 4.4% (P = 0.862) respectively. No statistically significant correlation was observed in the risk of post-operative surgical site infections between the four groups as shown in Table 2. Half of the patients 54.7% (n = 548) had a waiting time for admission of >8 h during all the different groups, with the highest number of surgeries 29.3% (n = 293) to be during group D (7:00 p.m. to 1:00 a.m.). The mean waiting time for admission to surgery was 11.0 ± 7.1 h in daytime group A and 10.8 ± 10.0 h for group B. In contrast, 11.8 ± 10.0 h and 11.1 ± 9.4 h were the mean waiting time for admission to surgery found in the nighttime groups, group C and D respectively. A statistically significant correlation (ordinal logistic regression analysis) was observed in the waiting time from admission to surgery between the four surgical groups (OR: 0.88, 95% CI: −9.2 to −8.1, P < 0.001) as shown in Table 2.

**Table 2:** Four shifts perioperative and postoperative outcomes in laparoscopic appendectomy.

Items	Group A	Group B	Group C	Group D	P-Value
	7.00 am–1.00 pm	1.00 pm–7.00 pm	7.00 pm–1.00 am	1.00 am–7.00 am	
Findings (N/%)					
Inflamed appendix	**266**/99.6%	**245**/99.4%	**194**/100%	**292**/99.7%	0.60
Mass	**21**/7.9%	**14**/5.4%	**12**/6.2%	**18**/6.1%	0.64
Abscess	**9**/3.4%	**7**/2.8%	**6**/3.1%	**8**/2.7%	0.97
Perforated	**13**/4.9%	**13**/5.3%	**16**/8.2%	**13**/4.4%	0.30
Gangrenous	**11**/4.1%	**9**/3.6%	**10**/5.2%	**14**/4.8%	0.86
Waiting time for admission (N/%)					<0.001
<1 h	**0**/0.0%	**0**/0.0%	**1**/0.5%	**2**/0.7%	
1–4	**59**/22.1%	**47**/19%	**44**/22.7%	**55**/18.8%	
5–8	**71**/26.6%	**57**/23.1%	**51**/26.3%	**66**/22.5%	
>8	**137**/51.3%	**143**/57.9%	**98**/50.5%	**170**/58.0%	
Mean (SD)	**11.0 (7.1)**	**10.8 (7.8)**	**11.8 (10.0)**	**11.1 (9.4)**	
Physicians					0.94
Residents	**147**/55.1%	**137**/55.5%	**102**/52.6%	**158**/53.9%	
specialist/fellow	**88**/33.0%	**80**/32.4%	**68**/35.1%	**91**/31.1%	
Consultants	**32**/12.0%	**30**/12.1%	**24**/12.4%	**44**/15.0%	
Complication (N/%)					
Bleeding	**2**/0.75%	**2**/0.81%	**2**/1.0%	**0**/0.0%	0.36
Bowel injury	**0**/0.0%	**0**/0.0%	**0**/0.0%	**0**/0.0%	NA
Surgical site infection	**3**/1.1%	**3**/1.2 %	**2**/1.0%	**5**/1.7%	0.77
Surgical site infection Location					0.83
Superficial	**0**/0.0%	**2**/67%	**1**/50%	**2**/40%	
Deep	**3**/100%	**1**/33%	**1**/50%	**3**/60%	
Surgical site infection treated by (N/%)					0.86
ABS only	**2**/67%	**3**/100%	**2**/100%	**2**/40%	
IRAb	**1**/33%	**0**/0.0%	**0**/0.0%	**2**/40%	
SXAB	**0**/0.0%	**0**/0.0%	**0**/0.0%	**1**/20%	
Surgery type					0.52
Drainage of umbilical collection	**0**/0.0%	**0**/0.0%	**0**/0.0%	**1**/0.34%	
Control of bleeding post-op	**1**/0.37%	**0**/0.0%	**0**/0.0%	**0**/0.0%	
Redo-surgery					
Yes	**1**/0.37%	**0**/0.0%	**0**/0.0%	**1**/0.34%	0.47
Re-admission	**2**/0.75%	**1**/0.40%	**2**/1.0%	**5**/1.7%	0.47
Conversion to laparotomy					
Yes	**1**/0.37%	**0**/0.0%	**0**/0.0%	**1**/0.34%	0.27
Hospital Stay (N/%)					<0.001
No stay	**16**/6.0%	**17**/6.9%	**15**/7.7%	**18**/6.1%	
1–3 days	**206**/77.2%	**194**/78.6%	**153**/78.9%	**223**/76.1%	
3–7 days	**42**/15.7%	**33**/13.4%	**25**/12.9%	**46**/15.7%	
>8 days	**3**/1.1%	**3**/1.2%	**1**/0.52%	**6**/2.1%	
Mean (SD)	**1.8 (1.5)**	**2.3 (1.7)**	**2.6 (1.8)**	**2.0 (1.5)**	
Mortality (N)					NA
Yes	**0**	**0**	**0**	**0**	

There was low incidence of post-operative site infections (SSIs) with 1.3% (n = 13, P = 0.77) in all groups (3 patients for group A (1.1%) and 3 patients (1.2%) during shift B, 2 patients for group B and 5 patients for group D), with almost all these patients having re-admission. Out of SSIs (5 patients, 0.5%) had superficial surgical site infection and (8 patients, 0.8%) had deep surgical site infection (postoperative intraabdominal) with no statistically significant difference between the four groups (P = 0.831). Among them, 9 patients (69.2%) were treated by antibiotics (ABS) (3 patients for daytime group B and 2 patients each for other groups A, C and D), 3 more patients were treated by IR drainage and antibiotics (IRAb) in (groups A and D) and 1 patient was treated by surgical drainage and antibiotics (SXAB) in (group D). The rest of the complications include: 6 patients (0.6%, P = 0.361) had post-op bleeding with one patient needing redo-surgery for bleeding control, and no patients had bowel injury. Regarding operating surgeon, 54.3% of the patients’ surgeries were performed by surgical trainee residents, 32.7% by fellows and 13.0% by attendings consultant, with no statistically significant difference among those in all four groups (P = 0.941). Only 2 patients (0.2%) in group C (7:00 p.m. to 1:00 a.m.) had conversions to laparotomy. Additionally, 2 patients from group A (for bleeding control) and D (for SSIs drainage), required a redo-surgery respectively. The mean length of hospital stay was found to be larger among those operated during the nighttime group C (2.6 ± 1.8, Table 2), and with statistically significant correlation (ordinal logistic regression analysis) between the four surgical groups (OR: 2.26, 95% CI: 0.75–0.93, P < 0.001). It is worth noting that 63.7% (n = 668) of patients have stayed at the hospital for 1–3 days. No mortality was reported among all surgical teams.

## 4 Discussion

Considering that appendectomy is one of the most performed operations by surgeons with many articles discussing the surgery itself, there is a few of publications discussing surgery outcomes or complications during different shift hours and whether the patients care gets impacted or not during the day or nighttime shifts. The present study demonstrates that the complication rates for laparoscopic appendectomy performed by surgeons in Hamad Medical Cooperation (HMC) during different day shift. The highest number of patient admission to the operation theatre was during the night shift (group D) at our institution. This could be explained by the fact that we are the main health care facility all over Qatar and we have a busy emergency department with significant numbers of patients admitted for surgical intervention. According to the priorities of cases and anesthesia recommendation, we prioritized more severe cases requiring major surgery, e.g., laparotomies, and more demanding laparoscopic cases, e.g., severely acute cholecystitis, and diagnostic laparoscopies in the morning shift. Also, the anesthesia team recommended starting with more complex cases in the morning time. For all these reasons, the main time for surgery for appendectomy was at the end of the day, except for complicated appendicitis that required urgent intervention. The findings of this study have significant ramifications for clinical practice. First and foremost, the findings are comforting as those complications did not seem to increase during night shift operations. We have established that appendectomy performed at night is linked with similar complications as appendectomy performed during the day.

In an observational study, Margenthaler et al. [28] reported a 1.8 percent death rate and a 16 percent rate of one or more complications. Additionally, Monttinen et al. [29] in another study found that out of 1,198 patients, there was a 4.8 percent complication rate and a 0.2 percent death rate. This study also had a relatively large sample size of 1001 patients. Of all patients, we had only 1.9 percent overall complication rate, and no patients experienced morbidity or mortality during the surgery, regardless of the time of day. Even the vast majority of patients across all groups had a hospital stay ranging from one to three days in length, there were few post-operative complications, with surgical site infection accounting for complication rate of 1.3 percent.

Frazee and Bohannon [30] reported a retrospective analysis of a total of 34 patients with gangrenous and perforating appendicitis, 7 percent and 42 percent of each study suffer from morbidity, respectively. According to a published article by Pier et al [31], 625 laparoscopic appendectomies were performed on 678 patients with suspected appendicitis, two percent of patients requiring conversion to laparotomies. In contrast, only 0.2 percent of patients in their study required open appendectomies.

The findings of a retrospective study by Nukta and co-workers and included 309 patients [20], showed that delaying appendectomies for acute appendicitis for 12–24 h after presentation does not significantly increase the rate of perforations, operative time, or hospital length of stay in patients diagnosed. In addition to optimizing the use of hospital resources such as nursing staff, anesthesia team, and surgical house staff during night shifts, it also reduced the interruption of the regular operating room schedule. Additionally, Omundsen and Dennett discovered no difference in complication rates or length of postoperative hospital stay between patients who underwent appendectomy within 12 h of admission and those who underwent appendectomy 12–24 h later [32]. The time of day when the surgery is performed may have an impact on the patient's decision-making, such as when to present to the hospital [33]. This demonstrates that the patient's time of presentation may be a significant issue to consider.

**Figure 1:**
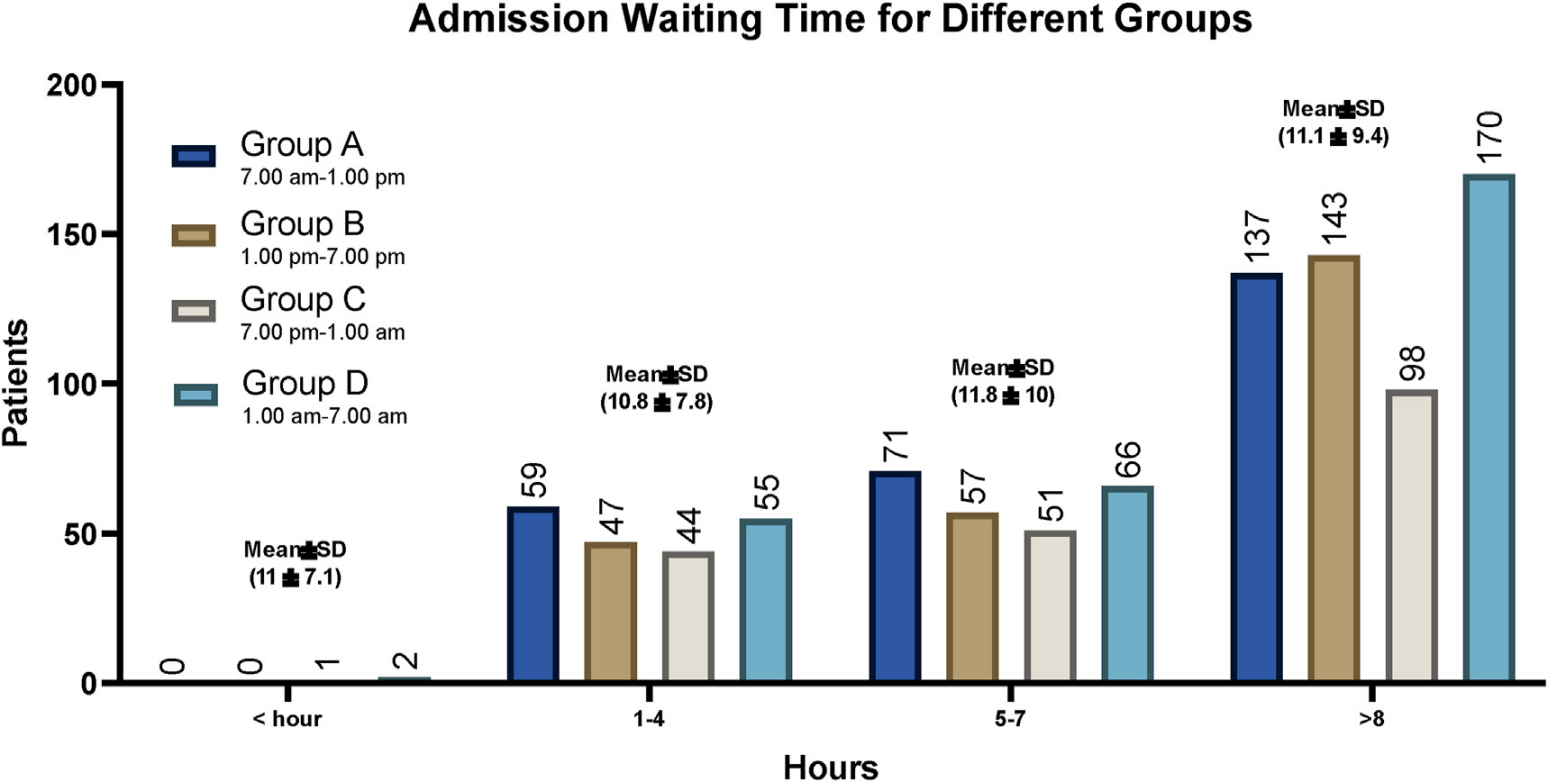
Four groups waiting time for admission in laparoscopic appendectomy.

In this work, most of our patients (54.7%) had more than 8 h of waiting time from admission to the operative room, while patients operated during nighttime group C had a slightly higher waiting time from admission to operative treatment as shown in Figure 1. This might be due to the fact that these patients were not having a perforation of the appendicitis and did not require emergency surgery. Another factor to consider is the dedication of theatre time to more complex cases in the morning time. A study reported by Drake et al. had found similar waiting time outcomes with no significant difference between the time of admission and complications, and longer waiting time for admission to the operative room did not show any perforation to the appendix [33].

**Figure 2:**
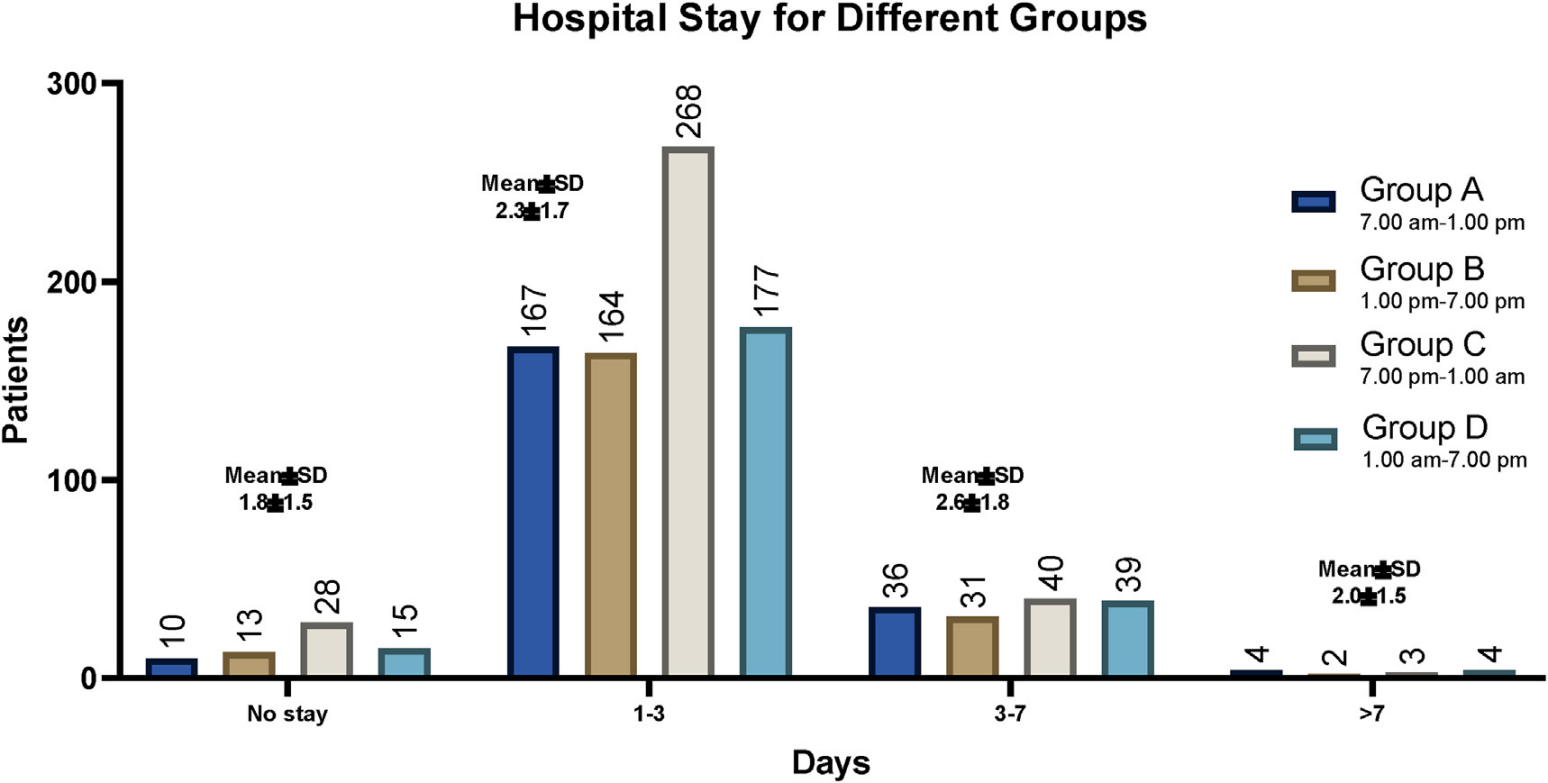
Four groups length of hospital stay in laparoscopic appendectomy.

In addition to the waiting times in our study, 77.5% of our patients stayed at the hospital for 1–3 days with a mean length of hospital stay to be larger among those operated during the nighttime group C (2.6 ± 1.8 days) as shown in Figure 2. In a similarly, the study by Lee and co-workers found that the average length of hospital stay was 3.7 ± 2.6 days [34].

The operative time during different shift hours is another point to look at in future work. Even though laparoscopic appendectomies are relatively a quick surgery, looking at operative time to see how long surgery takes to be completed and compare them during day and night may be an important factor to determine the efficacy of the surgery during different hours of the day. A study by Monttinen et al. showed that operations performed at night had a lower length of hospital stay [29]. Despite the increase in workload and sleepiness during the night shifts, a study by Tomasko and co-workers found that clinicians were able to perform surgeries proficiently and the rest of the staff were getting their tasks done correctly, and it also believes that operating at nighttime and having sleep deprivation does not decrease cognitive function but instead increases it [35, 36].

**Figure 3:**
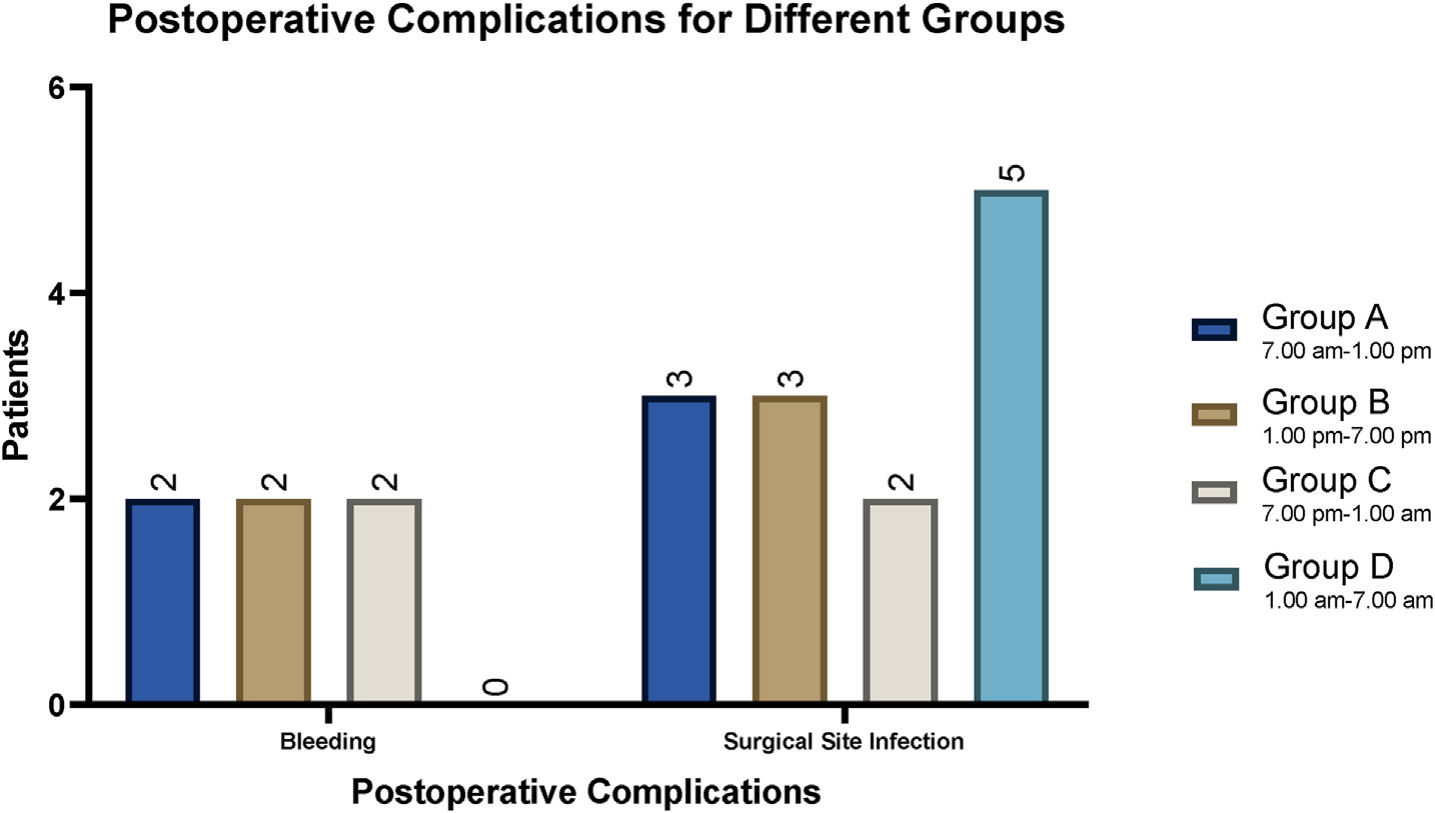
Four groups postoperative complications in laparoscopic appendectomy.

In this study, surgical skills for surgeons have also been evaluated between daytime and nighttime shifts and found that surgeons’ experience has no impact on postoperative outcomes as later were similar in the four groups (Figure 3). The most common postoperative complication was surgical site infection among all groups (13 patients, 1.3%), of which the majority were treated by ABS and risk was not correlated with different surgical groups.

We want to underscore that it is not always necessary to do appendectomies right away as it could be safer to postpone complicated operations sometimes if emergency surgery is not required. However, we believe that appendectomy can be performed safely at any time of day, regardless of the time of day it is performed. Furthermore, appendectomy is a rapid procedure, and in the event of an emergency requiring emergent surgery, it should not create a delay in the execution of these procedures.

The limitations of this study should be contemplated. This is a retrospective single-center study. However, the study has a relatively high number of patients, data are relatively comprehensive and peri- and postoperative clinical outcomes are carefully collected for all patients. More studies are still required with larger cohorts to shed more light in these findings.

## 5 Conclusion

In this study, we discovered that appendectomy conducted at nighttime operations are correlated with similar complications as appendectomy performed during the daytime, and that the varied shift hours had no effect on the complication rates or on the quality of care provided to patients at our hospital.

## Declarations

### Author contribution statement

Amjad A. Shah, Ahmad Zarour and Mohamed Said Ghali: Conceived and designed the experiments; Performed the experiments; Wrote the paper.

Raed M. Al-Zoubi: Conceived and designed the experiments; Performed the experiments; Analyzed and interpreted the data; Contributed reagents, materials, analysis tools or data; Wrote the paper.

Sharif Abdulzem, Khadija Qadir and Sara Sameer: Conceived and designed the experiments; Performed the experiments.

Ahmad R. Al-Qudimat, Mohamed Amine Rejeb and Laxmi Kumari Ojha: Analyzed and interpreted the data; Contributed reagents, materials, analysis tools or data; Wrote the paper.

### Funding statement

This research did not receive any specific grant from funding agencies in the public, commercial, or not-for-profit sectors.

### Data availability statement

Data included in article/supp. material/referenced in article.

### Declaration of interest's statement

The authors declare no conflict of interest.

### Additional information

No additional information is available for this paper.

## References

[1] Ferris M., Quan S., Kaplan B.S., Molodecky N., Ball C.G., Chernoff G.W. (2017). The global incidence of appendicitis. Ann. Surg..

[2] Coward S., Kareemi H., Clement F., Zimmer S., Dixon E., Ball C.G. (2016). Incidence of appendicitis over time: a comparative analysis of an administrative healthcare database and a pathology-proven appendicitis registry. PLoS One.

[3] Stewart B., Khanduri P., McCord C., Ohene-Yeboah M., Uranues S., Vega Rivera F. (2014). Global disease burden of conditions requiring emergency surgery. Br. J. Surg..

[4] Mandeville K., Monuteaux M., Pottker T., Bulloch B. (2015). Effects of timing to diagnosis and appendectomy in pediatric appendicitis. Pediatr. Emerg. Care.

[5] Carr N.J. (2000). The pathology of acute appendicitis. Ann. Diagn. Pathol..

[6] Alvarado A. (1986). A practical score for the early diagnosis of acute appendicitis. Ann. Emerg. Med..

[7] Andersson M., Andersson R.E. (2008 Aug). The appendicitis inflammatory response score: a tool for the diagnosis of acute appendicitis that outperforms the Alvarado score. World J. Surg..

[8] Paulson E.K., Kalady M.F., Pappas T.N. (2003). Suspected appendicitis. N. Engl. J. Med..

[9] Ergul E. (2007). Heredity and familial tendency of acute appendicitis. Scand. J. Surg..

[10] Michalinos A., Moris D., Vernadakis S. (2014). Amyand's hernia: a review. Am. J. Surg..

[11] Poprom N., Wilasrusmee C., Attia J., McEvoy M., Thakkinstian A., Rattanasiri S. (2020). Comparison of postoperative complications between open and laparoscopic appendectomy: an umbrella review of systematic reviews and meta-analyses. J. Trauma Acute Care Surg..

[12] Jaschinski T., Mosch C.G., Eikermann M., Neugebauer E.A., Sauerland S. (2018). Laparoscopic versus open surgery for suspected appendicitis. Cochrane Database Syst. Rev..

[13] Cao J., Tao F., Xing H., Han J., Zhou X., Chen T. (2017). Laparoscopic procedure is not independently associated with the development of intra-abdominal abscess after appendectomy: a multicenter cohort study with propensity score matching analysis. Surg. Laparosc. Endosc. Percutaneous Tech..

[14] Lau W.Y., Fan S.T., Yiu T.F., Chu K.W., Lee J.M. (1985). Acute appendicitis in the elderly. Surg. Gynecol. Obstet..

[15] Butler C. (1981). Surgical pathology of acute appendicitis. Hum. Pathol..

[16] Livingston E.H., Woodward W.A., Sarosi G.A., Haley R.W. (2007). Disconnect between incidence of nonperforated and perforated appendicitis: implications for pathophysiology and management. Ann. Surg..

[17] Kim K., Kim Y.H., Kim S.Y., Kim S., Lee Y.J., Kim K.P. (2012). Low-dose abdominal CT for evaluating suspected appendicitis. N. Engl. J. Med..

[18] van Dijk S.T., van Dijk A.H., Dijkgraaf M.G., Boermeester M.A. (2018). Meta-analysis of in-hospital delay before surgery as a risk factor for complications in patients with acute appendicitis. Br. J. Surgery.

[19] Pogorelić Z., Anand S., Žuvela T., Singh A., Križanac Z., Krishnan N. (2022 Jan 6). Incidence of complicated appendicitis during the COVID-19 pandemic versus the pre-pandemic period: a systematic review and meta-analysis of 2782 pediatric appendectomies. Diagnostics.

[20] Abou-Nukta F., Bakhos C., Arroyo K., Koo Y., Martin J., Reinhold R. (2006). Effects of delaying appendectomy for acute appendicitis for 12 to 24 hours. Arch. Surg..

[21] Eastridge B.J., Hamilton E.C., O'Keefe G.E., Rege R.V., Valentine R.J., Jones D.J. (2003). Effect of sleep deprivation on the performance of simulated laparoscopic surgical skill. Am. J. Surg..

[22] Teixeira P.G., Sivrikoz E., Inaba K., Talving P., Lam L., Demetriades D. (2012). Appendectomy timing: waiting until the next morning increases the risk of surgical site infections. Ann. Surg..

[23] Bickell N.A., Aufses A.H., Rojas M., Bodian C. (2006). How time affects the risk of rupture in appendicitis. J. Am. Coll. Surg..

[24] Blomqvist P.G., Andersson R.E.B., Granath F., Lambe M.P., Ekbom A.R. (2001). Mortality after appendectomy in Sweden, 1987-1996. Ann. Surg..

[25] Eldar S., Nash E., Sabo E., Matter I., Kunin J., Mogilner J.G. (1997). Delay of surgery in acute appendicitis. Am. J. Surg..

[26] Temple C.L., Huchcroft S.A., Temple W.J. (1995). The natural history of appendicitis in adults: a prospective study. Ann. Surg..

[27] Korndorffer J.R., Fellinger E., Reed W. (2010). SAGES guideline for laparoscopic appendectomy. Surg. Endosc..

[28] Margenthaler J.A., Longo W.E., Virgo K.S., Johnson F.E., Oprian C.A., Henderson W.G. (2003). Risk factors for adverse outcomes after the surgical treatment of appendicitis in adults. Ann. Surg..

[29] Mönttinen T., Kangaspunta H., Laukkarinen J., Ukkonen M. (2021). Nighttime appendectomy is safe and has similar outcomes as daytime appendectomy: a study of 1198 appendectomies. Scand. J. Surg..

[30] Frazee R.C., Bohannon W.T. (1996). Laparoscopic appendectomy for complicated appendicitis. Arch. Surg..

[31] Pier A., Götz F., Bacher C. (1991). Laparoscopic appendectomy in 625 cases: from innovation to routine. Surg. Laparosc. Endosc..

[32] Omundsen M., Dennett E. (2006). Delay to appendicectomy and associated morbidity: a retrospective review. ANZ J. Surg..

[33] Drake F.T., Mottey N.E., Castelli A.A., Florence M.G., Johnson M.G., Steele S.R. (2017). Time-of-day and appendicitis: impact on management and outcomes. Surgery.

[34] Lee J.M., Kwak B.S., Park Y.J. (2018). Is a one night delay of surgery safe in patients with acute appendicitis?. Ann. Coloproctol..

[35] Whelehan D.F., Alexander M., Connelly T.M., McEvoy C., Ridgway P.F. (2021). Sleepy surgeons: a multi-method assessment of sleep deprivation and performance in surgery. J. Surg. Res..

[36] Tomasko J.M., Pauli E.M., Kunselman A.R., Haluck R.S. (2012). Sleep deprivation increases cognitive workload during simulated surgical tasks. Am. J. Surg..

